# Within-Family Patterns of Sharing Instrumental Support to Older Parents of Multi-Child Families in China

**DOI:** 10.1093/geroni/igaa057.1654

**Published:** 2020-12-16

**Authors:** Jia Chen, Xiaochen Zhou

**Affiliations:** 1 Shanghai University, Shanghai, China; 2 University of Hong Kong, Hong Kong, China

## Abstract

In different multi-child families, adult children may share their instrumental support to older parents in distinct ways regarding its family mean level and differentiation among multiple offspring within families. Based on the family systems theory and the collective ambivalence perspective, we aimed (1) to identify different within-family patterns in relation to multiple offspring’s sharing instrumental support to an older parent in Chinese multi-child families; (2) to investigate potential individual and family predictors for different within-family patterns. Applying data from the China Family Panel Studies (2016, N=5791 older adults aged 60+), we employed latent profile analysis for classifying patterns and multinomial logistic regression for investigating predictors. Results showed three within-family patterns identified: independent (59.78%), highly-ambivalent (30.41%) and filial-cohesive (9.81%). Compared with the independent families, older parents in highly-ambivalent families were more likely to be older (OR=1.03), divorced/widowed (OR=0.61), to have lower educational levels(OR=0.84, ), poorer physical health (OR=0.92), to live in rural areas (OR=0.84), to have at least one adult daughter (OR=1.95)and one coresiding adult child (OR=3.22). Older parents in filial-cohesive families tended to be mothers (OR=0.82), divorced/widowed (OR=0.62), to have fewer adult children (OR=0.78) ,to have at least one adult daughter (OR=1.67) and one coresiding adult child (OR=2.16). The youngest adult children in filial-cohesive families tended to be older (OR=1.04). This study highlighted the importance of capturing different within-family dynamics regarding instrumental support to older parents among multiple adult children at the family level. It also uncovered the commons and differences between multi-child aging families in contemporary China.

